# The Effect of Acute High-Altitude Exposure on Oral Pathogenic Bacteria and Salivary Oxi-Inflammatory Markers

**DOI:** 10.3390/jcm13206266

**Published:** 2024-10-20

**Authors:** Pamela Pignatelli, Simona Mrakic-Sposta, Danilo Bondi, Domenica Lucia D’Antonio, Adriano Piattelli, Carmen Santangelo, Vittore Verratti, Maria Cristina Curia

**Affiliations:** 1 Marinaccad, MARINA NORD, 57127 Livorno, Italy; pamelapignatelli89p@gmail.com; 2 Institute of Clinical Physiology, National Research Council (IFC-CNR), 20162 Milan, Italy; simona.mrakicsposta@cnr.it; 3 Department of Neuroscience, Imaging and Clinical Sciences, University “G. d’Annunzio” Chieti—Pescara, 66100 Chieti, Italy; danilo.bondi@unich.it (D.B.); carmen.santangelo@unich.it (C.S.); 4 Department of Medical, Oral and Biotechnological Sciences, University “G. d’Annunzio” Chieti—Pescara, 66100 Chieti, Italy; dantoniodomenica@hotmail.com; 5 School of Dentistry, Saint Camillus International University of Health and Medical Sciences, 00131 Rome, Italy; apiattelli51@gmail.com; 6 Department of Psychology, University “G. d’Annunzio” Chieti—Pescara, 66100 Chieti, Italy; vittore.verratti@unich.it

**Keywords:** oral microbiota, saliva, altitude exposure, inflammation, periodontal status

## Abstract

**Background:** The environment can alter the homeostasis of humans and human microbiota. Oral health is influenced by high altitude through symptoms of periodontitis, barodontalgia, dental barotrauma, and a decrease in salivary flow. Microbiota and inflammatory state are connected in the oral cavity. This study aimed to explore the effect of acute high-altitude exposure on the salivary microbiome and inflammatory indicators. **Methods:** Fifteen healthy expeditioners were subjected to oral examination, recording the plaque index (PII), gingival index (GI), the simplified oral hygiene index (OHI-S), and the number of teeth; unstimulated saliva samples were collected at an altitude of 1191 m (T1) and 4556 m (T2). TNF-α, sICAM1, ROS, and the oral bacterial species *Porphyromonas gingivalis* (*Pg*) and *Fusobacterium nucleatum* (*Fn*) were quantified. **Results:** At T2, slCAM, TNF, and ROS increased by 85.5% (IQR 74%), 84% (IQR 409.25%), and 53.5% (IQR 68%), respectively, while *Pg* decreased by 92.43% (IQR 102.5%). The decrease in *Pg* was greater in the presence of low OHI-S. The increase in slCAM1 correlated with the reduction in *Fn*. Individuals with high GI and OHI-S had a limited increase in TNF-α at T2. **Conclusion:** Short-term exposures can affect the concentration of pathogenic periodontal bacteria and promote local inflammation.

## 1. Introduction

The living environment has a profound impact on the microbiome, whose composition and activity are malleable and influenced by environmental stressors. Among these stressors, hypoxia can favor the multiplication of anaerobic bacteria and collectively high altitude has been associated with increases in abundance of pro-inflammatory taxa, at least in the gut [1]. Instead, studies evaluating the effect of high altitude on oral samples are scarce, although the oral cavity is directly susceptible to external air. A recent study aiming at analyzing the microbiome from salivary samples obtained from orthodontic patients residing at high altitudes revealed a loss of microbiome diversity compared to sea level controls, due to the combined effect of hypoxia and a fixed orthodontic appliance [2]. Another study on altitude residents revealed that alpha diversity of the oral microbiota decreased, while beta diversity increased, with altitude, as previously reported regarding skin [3]. A very recent study investigated oral bacteriome from saliva and toothbrush samples of high-altitude-living Tibetans, finding differences among periodontal diseases in patients and healthy individuals who can rely on the adaptations to the harsh environment [4]. Oral health is indeed affected by high altitude: significant decrease in salivary flow, symptoms of acute pulpitis, periodontitis, gingivitis, and oral ulcers are the most common oral diseases reported during high-altitude travel; dental fluorosis, altered salivary flow, and periodontal inflammation can be cumulative in altitude residents, while barodontalgia and dental barotrauma only affect people who travel to altitudes [5].

The maintenance of oral health is contingent upon the equilibrium between the diverse microbial populations that inhabit the oral cavity. When this equilibrium is disrupted, dysbiosis is established. This condition arises due to the prevalence of certain pathogenic microorganisms over others and can be triggered by age, hormonal changes, antimicrobial agents, smoking, poor oral hygiene, or may be favoured by underlying pathologies. *Porphyromonas gingivalis* (*Pg*) is a Gram-negative anaerobic bacterium that has been identified as a periodontal pathogen and is associated with periodontitis [6]. This microorganism incites dysbiosis by impairing innate host defenses and can obstruct phagocytosis and promote inflammation, in particular oral inflammation, known as *Pg*-associated inflammation [7]. *Fusobacterium nucleatum* (*Fn*) is a Gram-negative anaerobe bacterium that is a common member of the oral microbiota. It has been associated with the formation of plaque biofilms and periodontitis [8,9].

Changes in the oral microbiota may be associated with periodontal status and external stressors such as altitude hypoxia. The growing interest in altitude traveling of the last decades raises the need for precise pathophysiological signatures to preserve travelers’ health. Microbiota and inflammatory status are closely interlinked into the oral cavity [10,11,12]. Dysbiosis can promote several oral diseases, including periodontal diseases and gingivitis [13], which are characterized by inflammation. Humans exposed acutely to hypoxia have hypoxic-inflammatory responses [14]. Pronounced immune-inflammatory responses are also present after high-altitude treks, along with an overproduction of reactive species and a reduction in antioxidant capacity, which collectively constitutes an OxInflammation (a dynamic and self-perpetuating vicious cycle between redox system and inflammation [15]) outbreak. Saliva represents an excellent source for obtaining biomarkers that are associated with pathological pathways; this comes with simplicity, and noninvasive sampling merits in comparison with other sources such as blood, and salivary biomarkers discovery is ever-growing [16]. Salivary biomarkers can serve for detecting non-invasively the molecular effects of hypoxia, as proteins involved in inflammatory and anti-bacterial properties of saliva, differentially expressed in healthy humans exposed for seven days to altitudes higher than 4000 m than normoxic controls [17]. Signaling pathways that modulate inflammation are detectable in saliva samples and can be associated with bodily responses and symptoms, as triggered by hypoxic exposure [18].

This study aimed to explore the impact of short-term high-altitude exposure on the salivary microbiome and inflammatory markers to understand whether this exposure can alter oral health status. In particular, the aim of this study was to analyze the composition of the microbiota and the state of OxInflammation in the oral cavity as a short-term response to high altitude, i.e., over 3000 m altitude within 24 h, which is common in altitude traveling.

## 2. Results

Oral indexes and visceral adipose area results are shown in Table 1. According to references, three participants had values of visceral fat over the recommended threshold [19] and two participants had poor oral hygiene [20].

**Figure 1:**
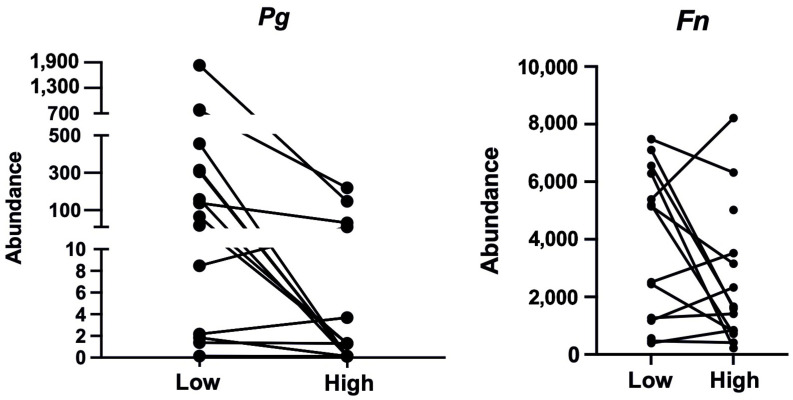
Individual changes of *Porphyromonas gingivalis* (*Pg*) and *Fusobacterium nucleatum* (*Fn*) from low to high altitude. Abundance is expressed as CFU/mL.

**Figure 2:**
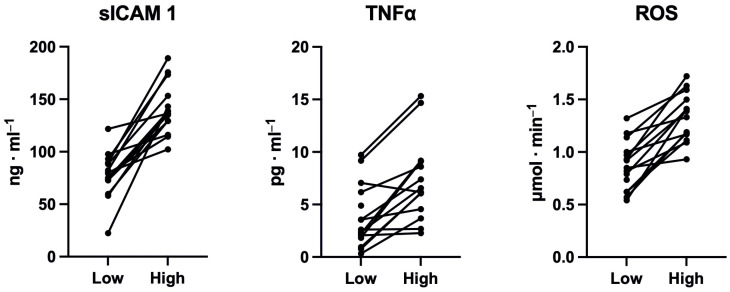
Individual changes of tumor necrosis factor alpha (TNF-α), soluble intercellular adhesion molecule-1 (sICAM1), and reactive oxygen species (ROS) from low to high altitude.

As reported elsewhere, SpO_2_ dropped and blood pressure increased as expected at high altitude; moreover, despite elevated leptin and diminished ghrelin levels, dietary intake was observed to be within the reference range for sports engagement and health, and dehydration did not occur [21]. Both salivary pH and osmolality did not change in response to the short-term trekking at high altitude [21]. At high altitude, *Pg* decreased by 92.43%, while slCAM, TNF, ROS increased by 85.5%, 84%, 53.5%, respectively. Instead, changes in *Fn* were more heterogeneous. Individual changes are shown in Figure 1 and Figure 2, and statistical results are included in Table 2.

The decrease in *Pg* was greater in the presence of low OHI-S (rho = 0.531 *p* = 0.065); the same occurred, although to a lower extent, in the presence of greater visceral fat (rho = −0.462 *p* = 0.115). The increase in sICAM correlated with the decrease in *Fn* (rho = −0.532 *p* = 0.078). Participants with high GI (rho = −0.693 *p* = 0.011) and OHI-S (rho = −0.544 *p* = 0.058) had a limited increase in TNFα at T2 (for individual values, see Figure S1).

## 3. Discussion

This study aimed to depict the salivary bacterial and inflammatory effects of short-term exposure to altitude hypoxia. Previous results existed on bacterial differences in high-altitude residents compared to normoxic residents. A significant amount of evidence has highlighted hypoxia-induced inflammation [22] and redox disturbances [23].

Unexpectedly, the hypoxic environment did not necessarily promote the proliferation of anaerobes into the oral cavity. Rather, we have shown that *Pg* abundance in saliva is reduced as a short-term response to high-altitude hypoxia (with a mean difference of 251.4 CFU/mL), while heterogeneous results were obtained for *Fn*. The 95% confidence interval of effect size ranged from 0.174 to 1.381 for low- vs. high-altitude paired comparison of *Pg* quantities, and the decrease was consistent across participants. These findings cannot be explained by changes in salivary osmolality, nor by changes in salivary acidity. Not even a good level of oral hygiene protected participants from changes in oral bacteria. Recently, Zhou and colleagues [24] reported that the relative abundance of genus *Porphyromonas* from unstimulated whole saliva was reduced after being exposed to an altitude of 4500 m. Those authors collectively stated that the saliva of the altitude group was found to contain a greater proportion of anaerobic bacteria, which were observed to inhibit the colonization of aerobic bacteria; however, the results obtained for *Pg* inform us that acute responses differ across species due to putative different pathways.

OxInflammatory response occurred in all participants, although to a different extent and represented by differential individual responses. Considering concentration of salivary TNFα poorly mirrors blood concentration in healthy adults [25], we can argue the presence of a local inflammation in oral cavity due to high-altitude stress. However, TNFα has been known to contribute to the chronicity of inflammatory diseases along with IL1β by activating the IL6-STAT3 pathway [26]. Participants with a high GI index and worse oral hygiene (high OHI-S) had a limited increase in salivary TNFα compared to those with reduced gingival inflammation. Probably, in these subjects the oral subclinical inflammation masked the OxInflammatory response. This effect was not mirrored by putative oral dysbiosis. This suggests that the effect is not necessarily due to microbiome pathways during acute response to hypoxia. It can be speculated that the OxInflammation in the oral cavity occurs early during altitude hypoxia exposure, and that changes in oral bacteria thus do not mirror the OxInflammatory pathways. However, hypoxia, cold, and collectively high altitude could induce an alteration of the oral microbial network even under healthy oral conditions; in fact, other studies show that the alpha diversity of the oral microbiota decreased in the high-altitude group (>4000 m) while beta diversity increased with altitude [3,27]. In addition to high-altitude residents, alpha diversity is also reduced in lowlanders after being exposed to high altitude [24].

While there is a decline in *Pg* concentration at elevated altitudes, this is not observed with *Fn*. The trend in *Fn* concentration greatly varies among participants exposed to hypoxia, likely due to the limited sample size. In vivo, pathobionts *Fn* and *Pg* are associated in the oral health status. Unlike *Pg*, *Fn* does not require a capnophilic environment and can support *Pg* survival in CO_2_-depleted environments [28]. A reduction in blood PaCO_2_ was observed at high altitude and it has already been found that the survival of *Pg* is reduced in monoculture with low CO_2_. Exposure to hypobaric hypoxia has been shown to induce an increased production of superoxide dismutase (SOD), which has the function of detoxifying with the formation of H_2_O_2_; this is in contrast to the effects observed in normobaric hypoxia [29].

The presence of elevated levels of SOD in the absence of corresponding levels of nicotinamide adenine dinucleotide reduced form (NADH)–oxidase/peroxidase activity may be detrimental to anaerobic microorganisms, potentially leading to the generation of significant quantities of H_2_O_2_ that cannot be effectively detoxified. This phenomenon may result in a reduced survival capacity of *Pg*. Conversely, *Fn* appears to benefit from a higher proportion of NADH oxidase/peroxidase [28]. The trend in *Fn* and *Pg* abundance could be related to the efficiency of the radical detoxification system, dependent on the balance between these two antioxidant enzymes.

It has been demonstrated that *Pg*, a keystone oral pathogen, requires receptors C5aR and C3aR (which increases C5aR expression) to survive and promote polymicrobial inflammatory diseases [30]. C5a and C3a are activated fragments of complement components 5 and 3, respectively, that serve as anaphylatoxins with oxidant-inducing and inflammatory-inducing properties, in particular C5a being the most potent anaphylatoxin of the complement system. Unlike other microbes, *Pg*, although an overall inhibition of the complement cascade, selectively generates the fragment C5a rather than C5b, which is proteolitically destroyed by the same bacterial species; in particular, *Pg* acts through the crosstalk between Toll-like receptor 2 (TLR2) and C5aR, thereby blunting the TLR2 antimicrobial response; *Pg* also degrades and inactivates C3a, preventing it from its bactericidal effect [31]. Hypoxia has been demonstrated to induce TLR2 expression via HIF-1α [32]. Therefore, we can speculate that hypoxia prevents the hijacking effect of *Pg* on antimicrobial defense, thus resulting in a reversed proliferation of this pathogen. The decrease in *Pg* was greater in individuals with good oral hygiene in a hypoxic environment, in accordance with the strong positive linear correlation between the amount of *Pg* and the OHI-S score in adults and elderly with periodontal disease [33].

Very severe hypoxia enhances the formation of ROS, furtherly exacerbated by bacterial inflammation; additive stressors of *Pg* metabolites and hypoxia (1% O_2_) have been demonstrated to exacerbate inflammation of periodontal tissues in vivo, through HIF-1 and NF-κB pathways [34]. An assessment of how less severe hypoxia in eubiosis affects these pathways in vivo would be intriguing.

Interventions to optimize the oral health of populations, according to ethnicity and living altitude, with tailored dental care strategies have been suggested, which also emphasizes the necessity of greater investigation into certain microbial pathways [4]. We herein state that short-term altitude exposure does not pose specific risks for increased abundance of the two oral pathobionts investigated, i.e., *Pg* and *Fn*. This result increases the need to depict a timeline of responses and chronic adaptations to high-altitude environments for different bacterial species linked to specific inflammatory pathways into the oral cavity.

This work did not come without limitations, as are common in high-altitude field studies. Our group of participants was a bit heterogeneous, both for anthropometric and inflammatory status, as well as for oral health. These plausible methodological biases stem from the ecological aspects of our high-altitude field study, which make it difficult to include a large sample size with homogeneous features by design. Nevertheless, none of the participants showed any signs of periodontitis or gingivitis, nor were they affected by peri-implantitis, nor were they undergoing treatments that target oral pathogens (laser applications and photodynamic therapy [35]). the consistent findings we obtained can be extended to longer days of exposure and larger samples, to identify the time course of responses while controlling for several confounding factors. This desirable extension would help in depicting the possible bias due to inter-day variability of bacterial species abundance. Other factors may account for the differences in oral bacterial abundance and oxi-inflammatory markers, as genetics and exposome, which would deserve focus in further studies.

## 4. Materials and Methods

### 4.1. Study Design and Participants

The project, entitled “Monte Rosa Exploration & Physiology”, was conducted between the 29th of August and the 2nd of September 2021 in the Western Alps of Italy. A group of 15 healthy expeditioners (8 men and 7 women, age range: 24 to 63 years) of the same ethnic group undertook a trek from Alagna Valsesia up to Capanna Regina Margherita, which is the highest European mountain lodge. Due to the nature of this study, calculation of minimum required sample size was not performed. Considering a two-tailed paired *t*-test with sample size of 15, α = 0.05 and 1 − β = 0.8, the minimum required effect size is d = 0.778 (calculations carried out using G*Power version 3.1 software). This methodological bias stems from the ecological aspects of our high-altitude field study, which make it difficult to include a large sample size with uniform features by design. Heart disease diagnosis, vascular illness, coronary heart disease, chronic obstructive pulmonary disease, mental disorders, drug addiction, neurodegenerative neurological diseases, respiratory failure, and oral inflammatory diseases were the exclusion criteria. None of the participants suffered from any oral inflammatory disease, nor were they using antibiotics and immunosuppressants, nor were they undergoing treatments that target oral inflammation as photodynamic therapy.

**Figure 3:**
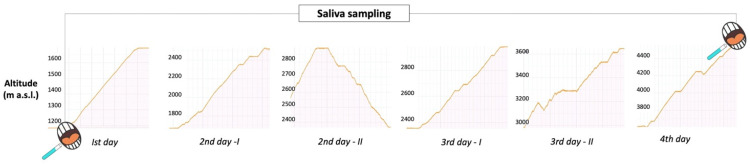
Study design of the “Monte Rosa Exploration and Physiology” project. Altitude was registered during the trek by using MeteoTracker portable station, whose online platform was used to create graphs.

The altitude profile is presented in Figure 3. All participants provided informed consent prior to their involvement in this study. This study was conducted as an ancillary project in accordance with the ethical standards set forth by the local ethics committee (Comitato Etico delle Province di Chieti e Pescara, protocol n.18, 29 July 2021). All participants were administered half a tablet of acetazolamide (125 mg) at an altitude of 2370 m, followed by a daily dose of one tablet (250 mg) at altitudes of 3647 and 4556 m. It should be noted that none of the participants exhibited any signs of periodontitis or gingivitis.

### 4.2. Clinical Variables Recorded and Sample Collection

During the oral examination, the plaque index (PI), gingival index (GI), and simplified oral hygiene index (OHI-S) were carried out by a specialist (author P.P., who is DDS, PhD, and oral surgeon). PI and GI were recorded using the standardized Oral Health Questionnaire, as previously reported [36]; briefly, PI and GI were measured on six surfaces on the Ramfjord teeth with a probe. OHI-S was evaluated on six dental surfaces (four posterior and two front teeth), recording the Debris score and the Calculus score using mirrors (MIR3HD, Hu-Friedy, Chicago, IL, USA), a dental probe (PCP-UNC 15, Hu-Friedy, Chicago, IL, USA), and an intra-oral light. An OHI-S value between 0.1 and 1.2 indicates good oral hygiene, between 1.3 and 3.0 moderate, and between 3.1 and 6.0 poor [20].

Visceral fat (VF) was estimated from Bioelectrical Impedance Analysis (BIA). Measurements were conducted by a specialist utilizing the HUMAN IM TOUCH multi-frequency analyzing device (DS MEDICA, Milan, Italy) with participants maintaining a supine position for a period of five minutes prior to the initiation of the test, with the legs and arms positioned at an angle of approximately 45° and 30°, respectively. Areas greater than 125 cm^2^ in men and 70 cm^2^ in women are associated with the cardiovascular risk factors of metabolic syndrome [19].

Unstimulated saliva samples were collected in the morning before breakfast from 15 healthy participants at altitude of 1191 m a.s.l. (Alagna Valsesia, Italy) and after 4 days at altitude of 4556 m (Capanna Regina Margherita, Monte Rosa, Italy). The collection entailed the allowing of drooling of patient’s naturally flowing saliva into 15 mL conical centrifuge tubes, ensuring the sterility of the collection instrument to avoid contamination. Before collection, participants were not allowed brush their teeth but were asked to drink water. Following the return of the samples to Alagna Valsesia, they were isothermally transported in boxes with ice packs to Chieti, and stored at −80 °C before the night.

### 4.3. Analyses of Bacterial Strains, DNA Extraction, and qPCR

Quantitative real-time polymerase chain reaction (qPCR) was used to quantify abundance of oral bacterial species *Porphyromonas gingivalis* (*Pg*) and *Fusobacterium nucleatum* (*Fn*). In this study, the reference bacterial strains *Fn* ATCC 25586 and *Pg* ATCC 33277 (LGC Standards S.r.l., Sesto San Giovanni, Milano, Italy) were used and cultured as reported previously [37]. Molecular analyses by qPCR were carried out to quantify the *Fn* and *Pg* abundances in each participant’s oral cavity [37].

Briefly, using a Quick DNA miniPrep Plus KIT (Zymo Research, Irvine, CA, USA), the entire genomic DNA of the reference bacterial strain and the samples were extracted. The quantities of *Pg* and *Fn* abundances in each sample were measured through a qPCR analysis using StepOne™ 2.0 (Applied Biosystems, Thermo Fisher Scientific, Waltham, MA, USA). The FadA gene for *Fn* was quantified using an assay based on Syber green. For *Pg*, the 16S rRNA (the gene encoding the small subunit of 16S ribosomal RNA) was quantified with a TaqMan Gene Expression Cstom assay (Life Technologies, ThermoFisher Scientific, Waltham, Massachusetts, USA).

A standard curve that shows the cycle threshold values in relation to the Pg 16SrRNA gene was created, passing through five points. By using this technique, we were able to calculate the number of bacteria based on the total amount of DNA that was extracted from the oral samples [37].

### 4.4. OxInflammation

Enzyme-linked immunosorbent assay (ELISA) technique was performed to quantify salivary tumor necrosis factor alpha (TNF-α, kit Cat. 589201, Cayman Chemical, Ann Arbor, MI, USA), and soluble intercellular adhesion molecule-1 (sICAM1, kit Cat. RAF102R, BioVendor, Brno, Czech Republic). The analyses were carried out in duplicate, and the concentrations were measured using spectrophotometry at a wavelength of 450 nm by comparing the optical density of the samples to standard curves; a microplate reader spectrophotometer (Infinite M200, Tecan Group Ltd., Männedorf, Switzerland) was used to read all the samples and standards, and the inter-assay coefficient of variation fell within the manufacturer’s specified range. The total production of reactive oxygen species (ROS) was measured using an X-band electron paramagnetic resonance spectroscopy (9.3 GHz) (E-Scan-Bruker BioSpin, Billerica, Massachusetts, USA) with spin probe CMH (1- hydroxy-3-methoxy-carbonyl-2,2,5,5-tetramethylpyrrolidine); the ROS determinations were converted into absolute quantitative values (μmol·min^−1^) using a stable radical, CP (3-carboxy2,2,5,5-tetramethyl-1-pyrrolidi-nyloxy), which was used as an external reference.

### 4.5. Statistical Analyses

Statistical comparisons were conducted using Jamovi (version 2.3.21) and GraphPad Prism (version 10.1.1) software, the latter also used to create the figures. Comparisons between low and high altitudes were conducted by t-test for paired two-tailed data; in particular, based on preliminary analysis of normality of residuals with Shapiro–Wilk test, either Student’s or Wilcoxon’s test was conducted. Statistical significance was set at *p* < 0.05; mean difference and effect size were computed and reported.

The percent differences in any values from low to high altitude were tested with non-parametric correlations in their associations with oral indexes and visceral fat. Statistical significance and rho coefficients were computed and reported.

## 5. Conclusions

Short-term exposures can influence the concentration of pathogenic periodontal bacteria and promote the state of local inflammation. For the first time, there is evidence that the hypoxic environment can reduce the presence of *Porphyromonas gingivalis*, at least in saliva. The insights from this study can foster the comprehension of pathophysiological pathways as linked to hypoxia and are translationally useful for targeting strategies aimed at improving travelers’ health.

## Figures and Tables

**Table 1:** Oral indexes and visceral fat summary data of participants.

	Median	IQR	Min	Max
***Plaque index***	1.205	0.438	0.580	1.610
***Gingival index***	0.545	0.305	0.110	1.080
***Oral hygiene index***	1.835	0.620	1.000	3.720
***N. of teeth***	28	2	23	32
***Visceral adipose area (cm* ^2^ *)***	47.04	48.33	5.490	290.2

**Table 2:** Paired samples comparisons of microbial and inflammatory data.

	Test	*p*	Mean Difference	95% CI		Effect Size	95% CI	
				*Lower*	*Upper*		*Lower*	*Upper*
***Pg***	Wilcoxon	0.017	251.4 CFU/mL	−11.20	513.9	r = 0.714		
**log_10_*Pg***	Student	0.011	1.243	0.333	2.152	d = 0.789	0.174	1.381
***Fn***	Student	0.074	1554 CFU/mL	−176.3	3283	d = 0.543	−0.051	1.118
**sICAM1**	Student	<0.001	−62.97 ng/mL	−81.29	−44.64	d = 1.984	−2.890	−1.054
**TNFα**	Student	<0.001	−3.569 pg/mL	−5.091	−2.046	d = 1.353	−2.075	−0.606
**ROS**	Student	<0.001	−0.456 μmol/min	−0.594	−0.318	d = 1.907	−2.789	−1.000

*Pg*: *Porphyromonas gingivalis*; *Fn*: *Fusobacterium nucleatum*; sICAM1: soluble intercellular adhesion molecule-1; TNFα: tumor necrosis factor alpha; ROS: reactive oxygen species.

## Data Availability

The data that support the findings of this study are available from the corresponding author upon reasonable request.

## Supplementary Materials

The following supporting information can be downloaded at: https://www.mdpi.com/article/10.3390/jcm13206266/s1, Figure S1: Descriptive features of participants and individual changes from low to high altitude.
