# Supplementary figures and images for: The Effect of Acute High-Altitude Exposure on Oral Pathogenic Bacteria and Salivary Oxi-Inflammatory Markers
Source: J Clin Med. 2024 Oct 20;13(20):6266. doi: 10.3390/jcm13206266 (PMC11508378; doi:10.3390/jcm13206266)

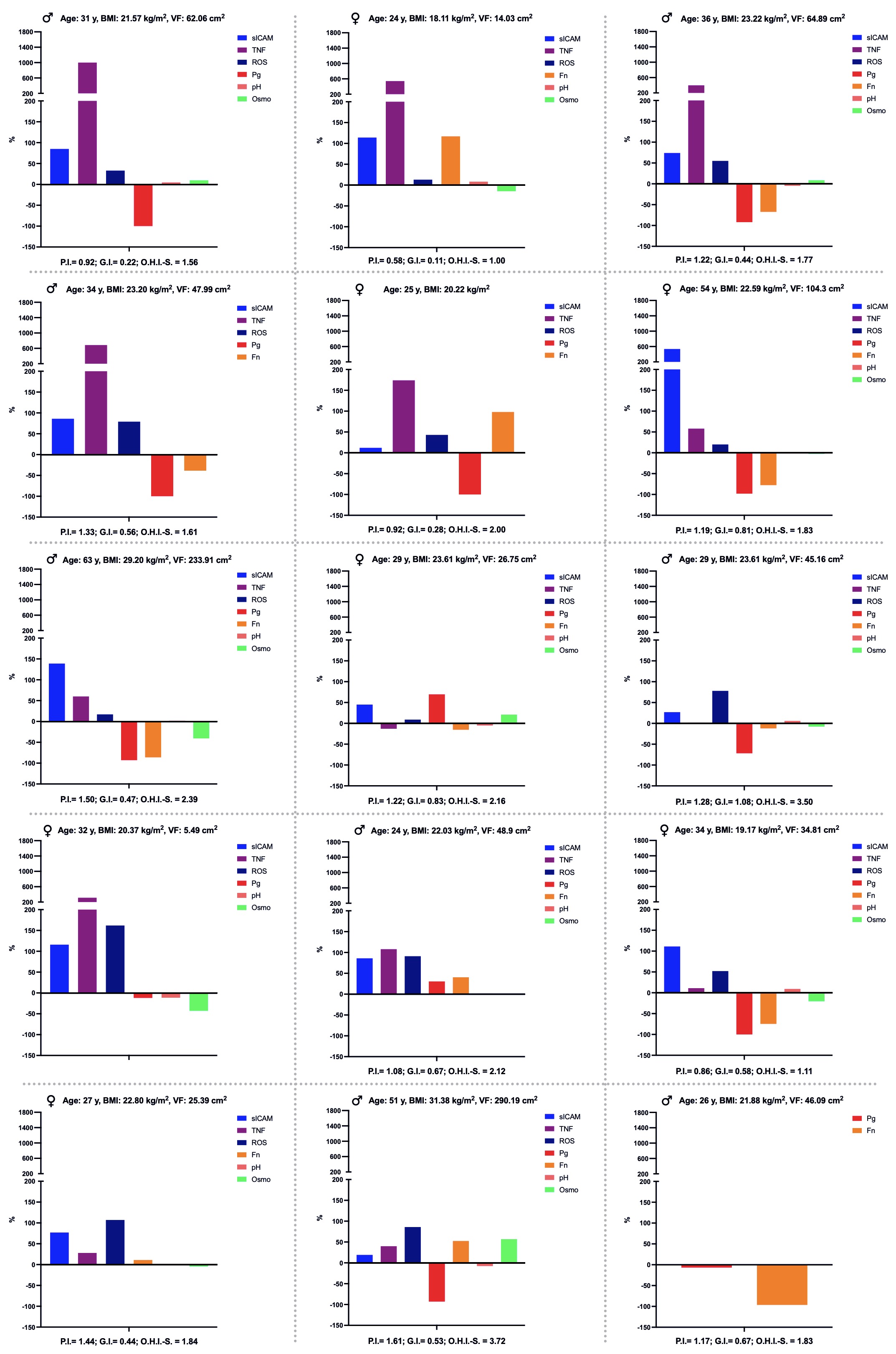

Supplement: Supplementary file 1 [file jcm-13-06266-s001.zip › jcm-3231836-supplementary.jpg]
